# Impact of Mechanical Ventilation and Anesthesia on PET Tracer Kinetics for Combined PET/fMRI Studies in Rats

**DOI:** 10.1007/s11307-025-02006-3

**Published:** 2025-05-14

**Authors:** Yan Ma, Laura Kuebler, Sabrina Haas, Andreas Maurer, Kristina Herfert

**Affiliations:** 1 Werner Siemens Imaging Center, Department of Preclinical Imaging and Radiopharmacy, University of Tuebingen, Tuebingen, Germany; 2 Max Planck Institute for Biological Cybernetics, Tuebingen, Germany; 3 Graduate Training Centre of Neuroscience, University of Tuebingen, Tuebingen, Germany; 4 Cluster of Excellence Ifit (EXC 2180) “Image Guided and Functionally Instructed Tumor Therapies”, University of Tuebingen, Tuebingen, Germany; 5 German Cancer Consortium (DKTK), DKFZ, Core Center Heidelberg, Heidelberg, Germany

**Keywords:** Positron emission tomography, Tracer kinetics, Mechanical ventilation, Anesthesia

## Abstract

**Purpose:**

Positron Emission Tomography (PET), a crucial tool in molecular brain imaging, has evolved into a hybrid system through integration with functional MRI (fMRI). This advancement facilitates the simultaneous recording of molecular and functional data in animal models, offering insights into neuroreceptor and neurotransmitter dynamics and their effects on brain function. While mechanical ventilation is often used in small animal fMRI to stabilize physiological blood gas levels, its effects on PET tracer kinetics remain underexplored.

**Procedures:**

This study examines the kinetics of [^11^C]raclopride, a dopamine-sensitive PET tracer targeting D2/D3 receptors, under various respiratory conditions and anesthesia protocols frequently used in small animal fMRI and PET.

**Results:**

Results indicate significant variations in tracer kinetics: increased peak levels, a shorter time to peak, and a faster tracer equilibrium in standard uptake value ratio were observed in spontaneously breathing animals versus those under mechanical ventilation. The anesthesia type also strongly influenced the tracer kinetics: α-chloralose anesthesia reduced brain uptake, whereas isoflurane led to a more rapid equilibrium.

**Conclusions:**

These findings underscore the profound impact of mechanical ventilation and anesthesia selection on PET tracer kinetics in hybrid PET/fMRI studies. The study highlights that those protocols established for fMRI are not directly transferable to PET imaging in small animals, emphasizing the necessity for a careful investigation of the influence of anesthesia and ventilation techniques on tracer kinetics.

**Supplementary Information:**

The online version contains supplementary material available at 10.1007/s11307-025-02006-3.

## Introduction

Positron emission tomography (PET) has evolved into the premier molecular imaging technique in clinical cancer diagnosis and preclinical research owing to its remarkable sensitivity. Its role has significantly expanded beyond oncology, with PET becoming an indispensable tool in neuroimaging, elucidating aspects such as glucose metabolism, oxygen consumption, and neurotransmitter distribution. The scarcity of methods to measure neuronal activity across the brain accentuates its relevance. Combining PET with other imaging modalities like computed tomography (CT) or magnetic resonance imaging (MRI) has enhanced its capability by compensating for its relatively low spatial resolution [1–3]. This integration facilitates concurrent anatomical and physiological imaging through CT, MRI and PET.

Moreover, the advent of functional MRI (fMRI) has led to the development of hybrid PET/MRI scanners that encapsulate the strength of both—molecular and functional—neuroimaging techniques. While fMRI primarily infers neural activity indirectly through hemodynamic responses, simultaneous PET/fMRI presents a unique advantage by merging the molecular insights from PET with the functional data from fMRI [4–10]. This integration has spurred global interest, with many laboratories applying PET inserts for simultaneous PET/MR small animal imaging.

However, integrating molecular and functional neuroimaging methods, such as specialized PET, fMRI, or their hybrid applications, presents unique challenges. One such challenge is developing acquisition protocols that accommodate the distinct principles of each modality. In small animal studies, selecting a suitable anesthesia protocol is crucial for consistent results interpretation across imaging techniques. Some researchers have performed neuroimaging in conscious animals to circumvent potential anesthesia-related issues, using physical restraints in awake animals [11, 12]. However, challenges such as stress from restraint and involuntary motion persist, complicating data analysis and interpretation in such setups, making anesthesia a common practice in small animal brain imaging.

The conventional small animal PET imaging protocol involves isoflurane inhalation at a concentration between 1.7–2.5% with spontaneous breathing in pure oxygen used as the delivery gas to prevent hypoxia [13, 14]. However, in simultaneous PET/fMRI research, such protocols must be cautiously applied, considering that high isoflurane doses can alter brain function, and pure oxygen can impact the blood-oxygen-level-dependent (BOLD) signal [15]. For fMRI studies in anesthetized rats, common practices include the use of pancuronium bromide to minimize motion artifacts and mechanical ventilation with air or oxygen-enriched air as the delivery gas [16, 17]. This approach helps to maintain stable arterial oxygen (pO_2_) and carbon dioxide (pCO_2_) levels, influencing cerebral blood flow (CBF), as they are major components of the BOLD signal. The impact of anesthesia and respiratory conditions on the BOLD signal has been extensively explored in small animal fMRI [18, 19], but has so far received less attention in small animal PET imaging [20, 21]. One widely used approach in small animal fMRI studies is low-dose isoflurane paired with mechanical ventilation. Isoflurane, a volatile anesthetic, is favored for its rapid induction and easy adjustability, allowing fine-tuned control of anesthesia depth. When combined with mechanical ventilation, it helps maintain stable physiological parameters, including blood gases and pH, reducing the risk of hypercapnia and hypoxia. However, its impact on cerebral blood flow and metabolic rates requires careful monitoring, as these factors can influence neuroimaging outcomes. Another protocol involves a combination of very low-dose isoflurane (0.5%) with the alpha- 2 adrenergic agonist medetomidine [6, 22]. This combination allows for reduced isoflurane concentrations, which mitigates its vasodilatory effects, while medetomidine provides sedative, analgesic, and muscle-relaxing properties. It offers a more stable hemodynamic profile, preserving cerebral blood flow and minimizing fluctuations in brain activity, making it particularly advantageous for longer imaging sessions where consistent physiological stability is crucial. Alpha-chloralose is also commonly utilized due to its minimal interference with neural activity and cerebral blood flow [23]. It induces a stable, long-lasting state of anesthesia with minimal cardiovascular effects, providing an advantage for functional studies that require minimal suppression of spontaneous neuronal activity. However, its administration is more complex and often requires precise dosing to achieve the desired level of sedation without compromising the physiological state of the animal.

Nevertheless, respiratory conditions or the use of anesthetics might substantially influence PET tracer kinetics, which are crucial for accurate data quantification and interventional studies. PET imaging often requires the tracer to reach early equilibrium, particularly in studies involving pharmacological, sensory, or optogenetic stimulations. This study aims to investigate the kinetics of [^11^C]raclopride under respiratory and anesthetic conditions typically employed in small animal fMRI studies. Five conditions were tested in this study. We anesthetized rats using a combination of 0.5% isoflurane and medetomidine, dividing them into three groups: one with spontaneous breathing, another with mechanical ventilation, and a third undergoing mechanical ventilation with continuous pancuronium bromide infusion for immobilization. Additionally, this study explores the effects of another two anesthetics in small animal brain imaging studies on [^11^C]raclopride kinetics. Specifically, 1.3% isoflurane was employed and a bolus plus constant infusion of α-chloralose was also evaluated.

## Material and Methods

### Animals

All animal experiments complied with the European Communities Council Directive (2010/63EU) and the German Animal Protection Law and were approved by the Tübingen Animal Protection Committee (Regierungspräsidium Tübingen, Germany). The rats were housed in groups of 4 with a 12 h light/dark cycle at a temperature of 22 °C and relative humidity between 40 and 60%. Food and water were obtainable ad libitum. This research used 20 female and 4 male Sprague Dawley rats (286.5 ± 36.8 g) purchased from Charles River Laboratories (Sulzfeld, Germany). For an overview of the different experimental groups please see Table 1.

**Table 1:** Experimental groups and [^11^C]raclopride injected and molar radioactivities at the time of injection

Anesthesia	Abbreviation	Group size	Injected activity [MBq]	Molar activity [GBq/µmol]
0.5% isoflurane and medetomidine – ventilation—pancuronium bromide	ISO+MED-V-P	*n* = 4	46.6 ± 1.8	84.0 ± 23.2
0.5% isoflurane and medetomidine + ventilation—pancuronium bromide	ISO+MED+V-P	*n* = 4	49.3 ± 1.7	84.0 ± 32.2
0.5% isoflurane and medetomidine + ventilation + pancuronium bromide	ISO+MED+V+P	*n* = 4	53.3 ± 4.4	116.8 ± 31.6
1.3% isoflurane + ventilation + pancuronium bromide	ISO+V+P	*n* = 4	52.0 ± 2.9	120.1 ± 36.9
α-chloralose + ventilation + pancuronium bromide	AC+V+P	*n* = 4	51.3 ± 7.1	98.6 ± 29.3

### Experimental Design

[^11^C]raclopride PET measurements were performed under three different respiratory conditions and anesthesia protocols to determine the influence on [^11^C]raclopride kinetics.

To investigate the impact of mechanical ventilation on the [^11^C]raclopride kinetics, all the rats were anesthetized with 0.5% isoflurane and medetomidine (absolute bolus 0.08 mg/kg and 0.15 mg/kg/h ip infusion). The rats were divided into the following groups:

1. no ventilation nor pancuronium bromide infusion (MED-V-P).
2. with ventilation and no pancuronium bromide infusion (MED+V-P).
3. with ventilation and pancuronium bromide infusion (MED+V+P).

To determine the effect of anesthetics on the [^11^C]raclopride kinetics, two additional anesthesia protocols were investigated. Both of these were paired with mechanical ventilation and pancuronium infusion:

(4) 1.3% isoflurane (ISO+V+P).
(5) α-Chloralose initiated with an absolute bolus of 42 mg/kg, followed by an infusion rate of 20 mg/kg/h ip infusion (AC+V+P).

Group 3, 4 and 5 received a constant ip infusion of pancuronium bromide of 1 mg/kg/h. Animals were ventilated with a breathing machine with 60 breaths/min of respiratory rate, 60% of inspiration and 0.5 ml/min of air in groups 2, 3, 4, and 5.

### Radiotracer Synthesis

[^11^C]Raclopride was synthesized as previously described [24, 25] (details are provided in the supplemental information).

### Animal Preparation and [^11^C]raclopride PET Imaging

PET imaging was performed on a dedicated small animal Inveon PET scanner (Siemens Healthcare, Knoxville (TN), USA). The rats were placed in a knock-out box, and 3% isoflurane evaporated in regular air was delivered for anesthesia induction through the small animal veterinary anesthesia machine (Landmark Anesthesia System, Vetland, Louisville (KY), USA). Once anesthetized, each rat weight was recorded, and the isoflurane concentration was reduced to 2% for subsequent procedures.

A 30 G needle catheter was inserted into a tail vein for the [^11^C]raclopride bolus injection. A second catheter was placed intraperitoneally for the constant infusion of the corresponding anesthetics (MED-V-P, MED+V-P, MED+V+P and AC+V+P). All groups except MED-V-P were endotracheally intubated using a 14 G plastic tube and connected to a small animal ventilator (DC1 73–3629, Harvard Apparatus, Holliston, MA, USA) connected to isoflurane and air. The respiration rate was set to a constant rate of 60 breaths per minute.

Rats were then positioned in the center of the PET scanners field of view on carbon beds with a water heated mat. The temperature of the rats was constantly monitored using a rectal probe and maintained at about 37 °C by a temperature feedback-controlled system (Medres, Cologne, Germany). At least thirty minutes prior to the PET acquisition, the isoflurane concentration was reduced to 1.3% (ISO+V+P), 0.5% (MED-V-P, MED+V-P, MED+V+P), and 0% (AC+V+P) in regular air and kept constant during the whole time-course of the experiment.

[^11^C]Raclopride was injected intravenously as a fast bolus using a computer-controlled infusion pump (Harvard Apparatus PHD 2000 Infusion Pump) at a speed of 1000 µl/min for 30 s with an activity concentration of 100 MBq/ml. Dynamic PET data acquisition was started 5 s before tracer injection and was continued for 60 min followed by a 13-min transmission measurement with a cobalt- 57 point source for attenuation correction. Dynamic PET data was divided into 39 time-frames (12 × 5 s, 6 × 10 s, 6 × 30 s, 5 × 60 s, 10 × 300 s). Images were reconstructed using a 3D ordered subset expectation maximization (OSEM) algorithm using a matrix size of 128 × 128 × 63 resulting in a voxel size of 0.388 × 0.388 × 0.796 mm^3^.

### Image Analysis

PET image analysis was carried out using PMOD 4.2 software (PMOD Technologies, Zürich, Switzerland). To obtain quantitative images, dead time, decay correction and normalization were applied. To avoid motion during the PET imaging session, the head was fixed with removable tape. PET images were aligned to a reference MR rat brain atlas with volumes of interest (VOI) matching the atlas [26]. Time activity curves (TACs) of both the left and right caudate putamen (CPu) and the cerebellum were derived and converted into standardized uptake values (SUVs), calculated using the following equation:

$$SUV \left(t\right)= \frac{radioactivity\;concentration (\frac{kBq}{mL})}{injected\;dose/animal\;weight (\frac{kBq}{g})}$$

The SUV ratio was then calculated as SUVR- 1 from individual TACs using the cerebellum (CER) as a receptor-free reference region in the following equation:

$$SUVR-1 = \frac{ SUV\;CPu}{SUV\;CER}-1$$

The peak [^11^C]raclopride activity, as well as the time taken to reach this peak in the CPu and cerebellum, were obtained from the TACs. The delivery rate of the tracer was determined by the quotient of the peak [^11^C]raclopride activity and the time to peak values.

### Statistical Analysis

Data followed a normal distribution, comparisons were performed by one-way ANOVA with false discovery rate (FDR) correction (see supplementary methods for details).

## Results

### Standard PET Imaging Protocol

**Fig. 1:**
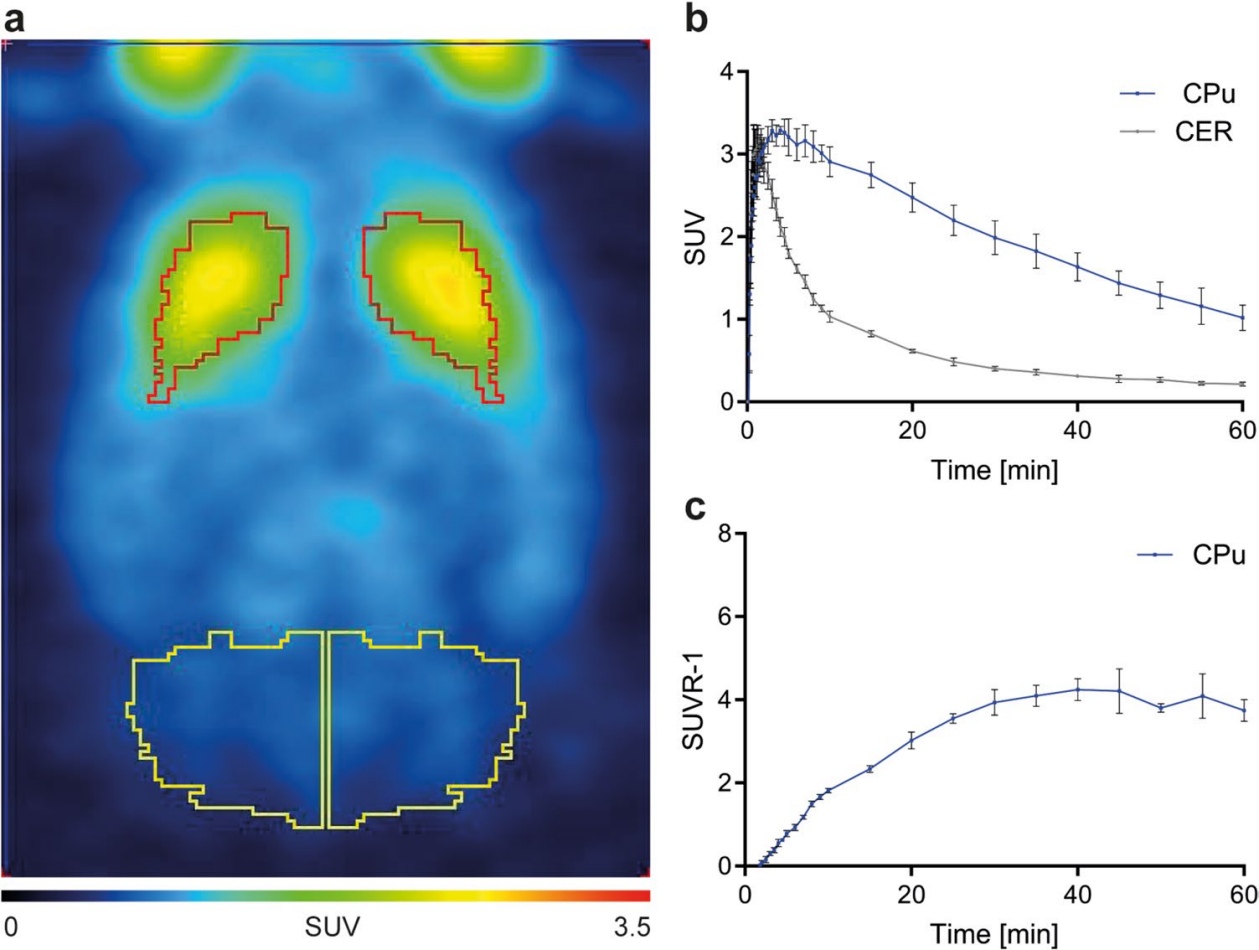
Pharmacokinetic and pharmacodynamic profile of [^11^C]raclopride in healthy rats under 1.7–2.5% isoflurane in 100% O_2_. **a** Normalized rat brain PET/MR image (mean of four rats) summed up over 60 min with regions of interest in the caudate putamen (red) and cerebellum (yellow). **b** Corresponding time-activity curves of [^11^C]raclopride in the caudate putamen and cerebellum as well as the (**c**) SUVR- 1 in the caudate putamen. Abbreviations: CPu: caudate putamen; CER: cerebellum; SUV: standard uptake value; SUVR- 1: standard uptake volume ratio- 1

Figure 1 shows the normalized brain PET images, decay-corrected TAC, and corresponding SUVR- 1 values for [^11^C]raclopride under a standard PET imaging protocol. The rats were anesthetized with isoflurane at a concentration of 1.7%, with spontaneous breathing maintained in pure oxygen. Figure 1b and c illustrate the brain kinetics following bolus injection of [^11^C]raclopride, demonstrating that pseudo-equilibrium between the striatum and cerebellum is reached within 30 min post-injection.

### Effect of Respiratory Conditions on [^11^C]raclopride Kinetics

**Fig. 2:**
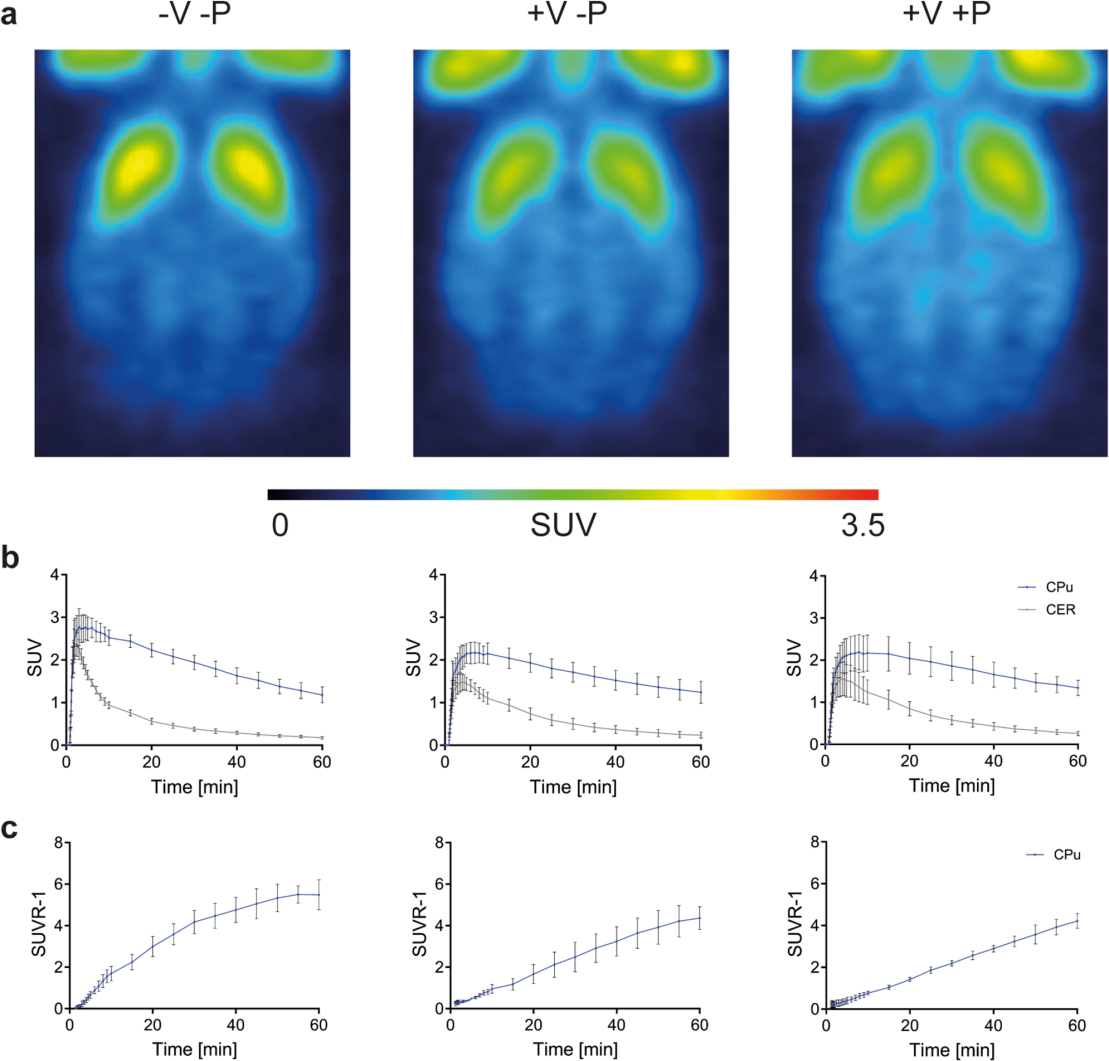
Effect of mechanical ventilation on [^11^C]raclopride kinetics under isoflurane and medetomidine anesthesia. **a** Normalized rat brain PET images (mean of four rats) are illustrated summed up over 60 min from group 1 (-V-P), group 2 (+V-P) and group 3 (+V+P). **b** [^11^C]Raclopride time activity curves of group 1, group 2 and group 3 (left to right) are plotted over time for the caudate putamen and the cerebellum. **c** Corresponding binding values calculated as SUVR- 1 over time are shown. Abbreviations: CPu: caudate putamen; CER: cerebellum; SUVR- 1: distribution volume ratio- 1; SUV: standard uptake value; V: ventilation; P: pancuronium bromide

The normalized brain PET images (Fig. 2a) are illustrated as sum of all frames from MED-V-P, MED+V-P and MED+V+P. Figure 2b displays the mean decay-corrected TACs of [^11^C]raclopride in the CPu and cerebellum post-bolus injection and the corresponding SUVR over time for MED-V-P, MED+V-P, MED+V+P. Rats of MED-V-P demonstrated a rapid brain uptake of [^11^C]raclopride with a peak SUV of 2.77 ± 0.43 in the bilateral CPu and a peak SUV of 2.36 ± 0.28 in the cerebellum. In contrast, MED+V-P and MED+V+P exhibited a lower peak SUV in the bilateral CPu: 2.17 ± 0.25 for MED+V-P and 2.19 ± 0.42 for MED+V+P. The peak SUV of the cerebellum was 1.48 ± 0.20 for MED+V-P and 1.55 ± 0.40 for MED+V+P.

By the 50-min mark following [^11^C]raclopride injection, equilibrium was achieved between the CPu and the cerebellum using MED-V-P. However, neither MED+V-P nor MED+V+P reached this state of equilibrium, as depicted in Fig. 2c.

**Fig. 3:**
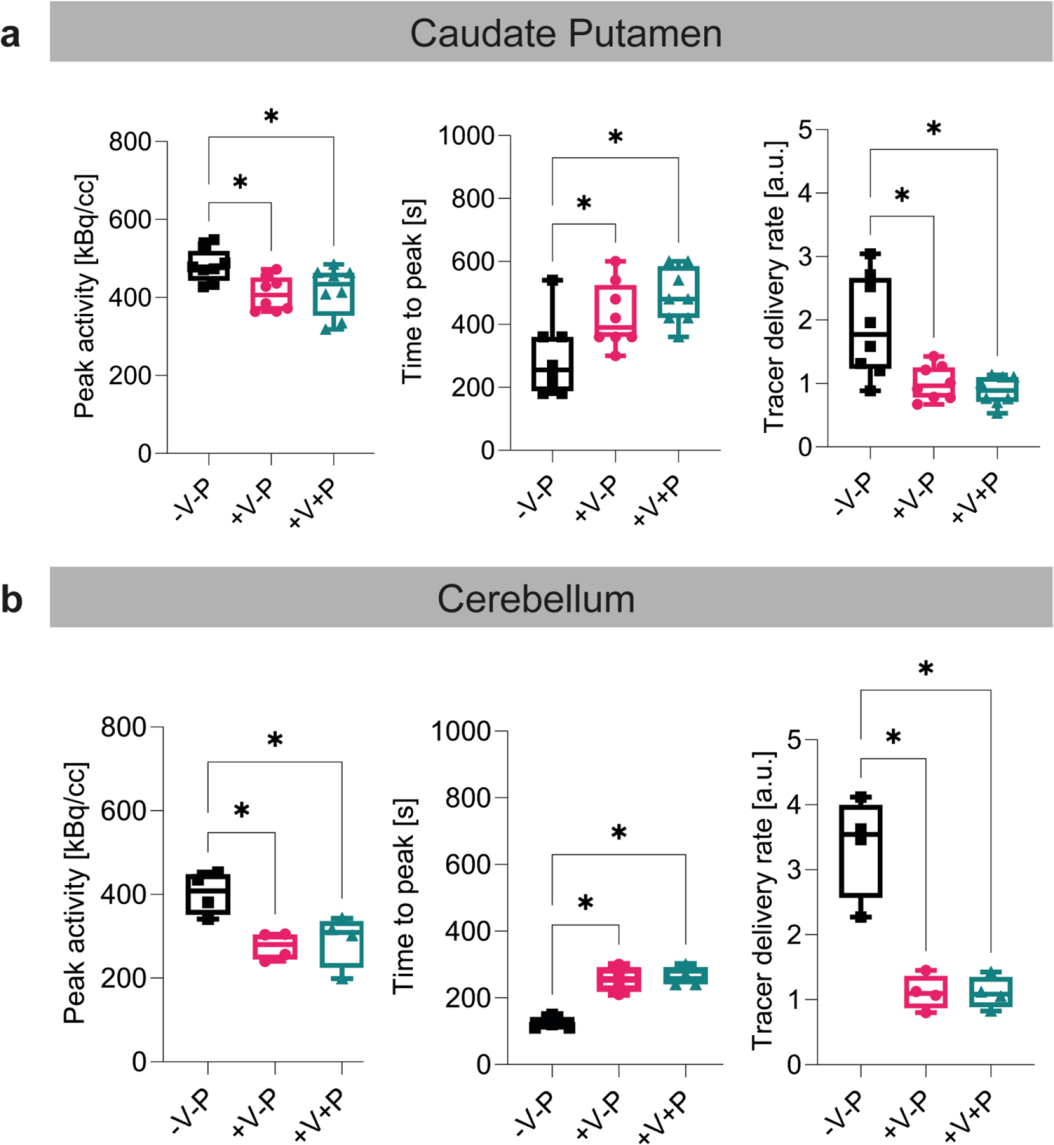
Effect of respiratory conditions on the delivery rate of [^11^C]raclopride under 0.5% isoflurane and medetomidine. **a** Peak activity, time to peak and tracer delivery rate of [^11^C]raclopride illustrated as box plots indicating the mean and standard deviation of the caudate putamen and **b** the cerebellum for group 1 (-V-P), group 2 (+V-P) and group 3 (+V+P). *p ≤ 0.05. Tracer delivery rate = Peak activity/Time to peak. Abbreviations: V: ventilation; P: pancuronium bromide

Maximal [^11^C]raclopride activity, time to peak, and tracer delivery rate of [^11^C]raclopride for the CPu and the cerebellum under different respiratory conditions using 0.5% isoflurane and medetomidine are illustrated in the Fig. 3 and Table S1. Specifically, rats in MED-V-P, who were breathing spontaneously, displayed a markedly elevated peak [^11^C]raclopride activity, a reduced time to peak, and consequently a faster delivery rate in the CPu and cerebellum compared to ventilated animals in MED+V-P and MED+V+P. Interestingly, no difference was observed in the kinetics of [^11^C]raclopride between the ventilated rats of MED+V-P and MED+V+P, regardless of whether they received pancuronium bromide infusion or not. For more detailed results, refer to the supplementary material.

### Effect of Different Anesthetics on [^11^C]raclopride Kinetics

**Fig. 4:**
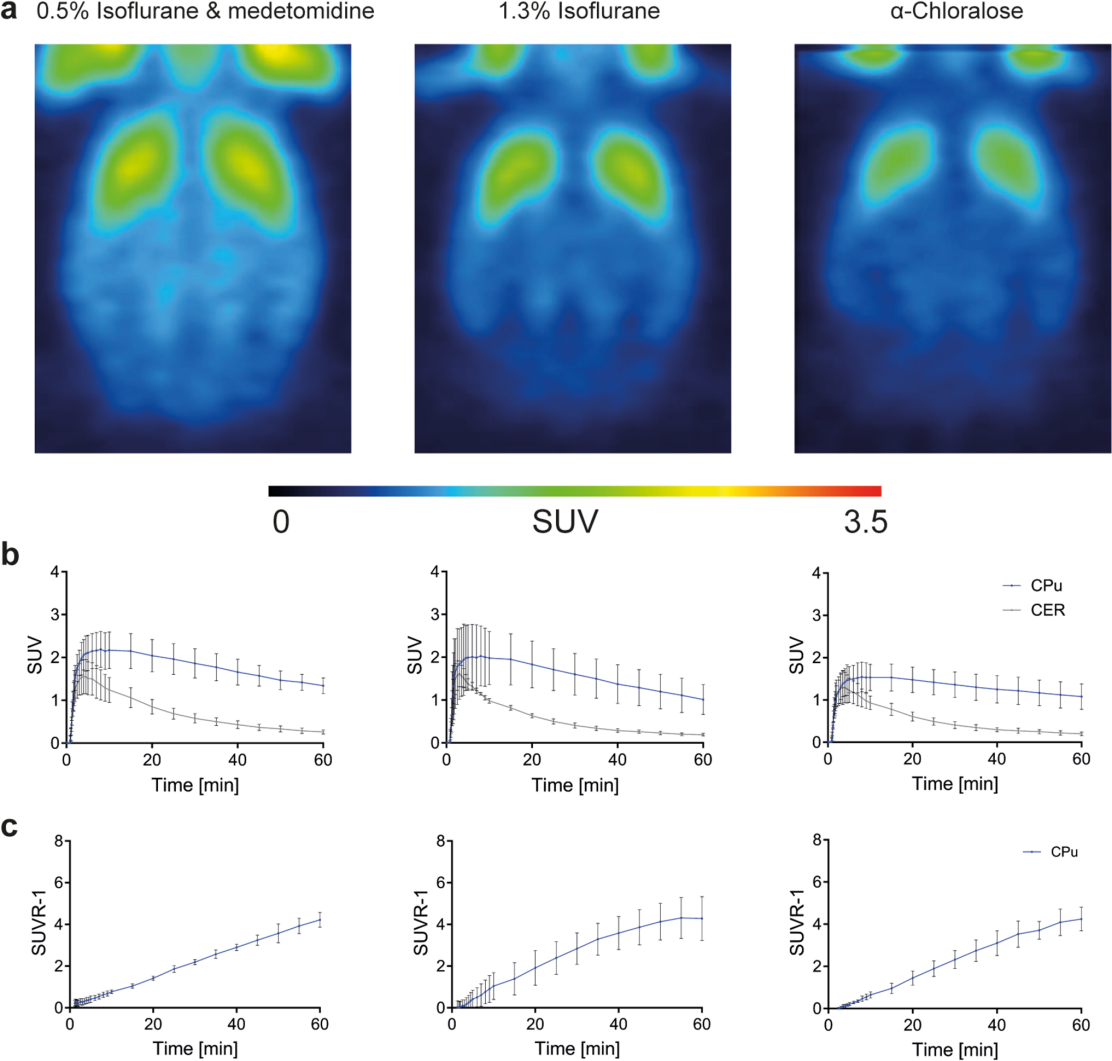
Effect of anesthesia with ventilation and pancuronium infusion on [^11^C]raclopride kinetics. **a** Normalized rat brain PET images of group 3 (0.5% isoflurane and medetomidine), group 4 (1.3% isoflurane) and group 5 (α-chloralose) (mean of four rats) are illustrated summed up over 60 min. **b** [^11^C]Raclopride time activity curves are plotted over time for the caudate putamen and the cerebellum. **c** Corresponding binding values calculated as SUVR- 1 over time are shown. Abbreviations: CPu: caudate putamen; CER: cerebellum; SUVR- 1: standard uptake volume ratio- 1; SUV: standard uptake value

In subsequent experiments, we evaluated the TACs of [^11^C]raclopride in the CPu and cerebellum and the corresponding SUVR- 1 using two alternative standard anesthesia protocols, ISO+V+P and AC+V+P, while maintaining both mechanical ventilation and pancuronium bromide infusion (Fig. 4). The normalized brain PET images (Fig. 4a) are illustrated as sum of all frames from MED+V+P, ISO+V+P and AC+V+P. Figure 4b displays the mean decay-corrected TACs of [^11^C]raclopride in the CPu and cerebellum post-bolus injection and the corresponding SUVR- 1 over time. The TACs of MED+V+P and ISO+V+P exhibited heightened peak SUV values in the bilateral CPu of 2.19 ± 0.42 for MED+V+P and 2.03 ± 0.70 for ISO+V+P. For the cerebellum, the peak SUVs were 1.55 ± 0.40 for MED+V+P and 1.61 ± 0.30 for ISO+V+P. However, AC+V+P showed lower peak SUV values of 1.54 ± 0.32 in the unilateral bilateral CPu and 1.29 ± 0.40 in the cerebellum.

This consistency, particularly evident in MED+V+P from the initial experiment, suggests that the impact of ventilation on tracer kinetics manifests independently of the type of anesthesia employed.

**Fig. 5:**
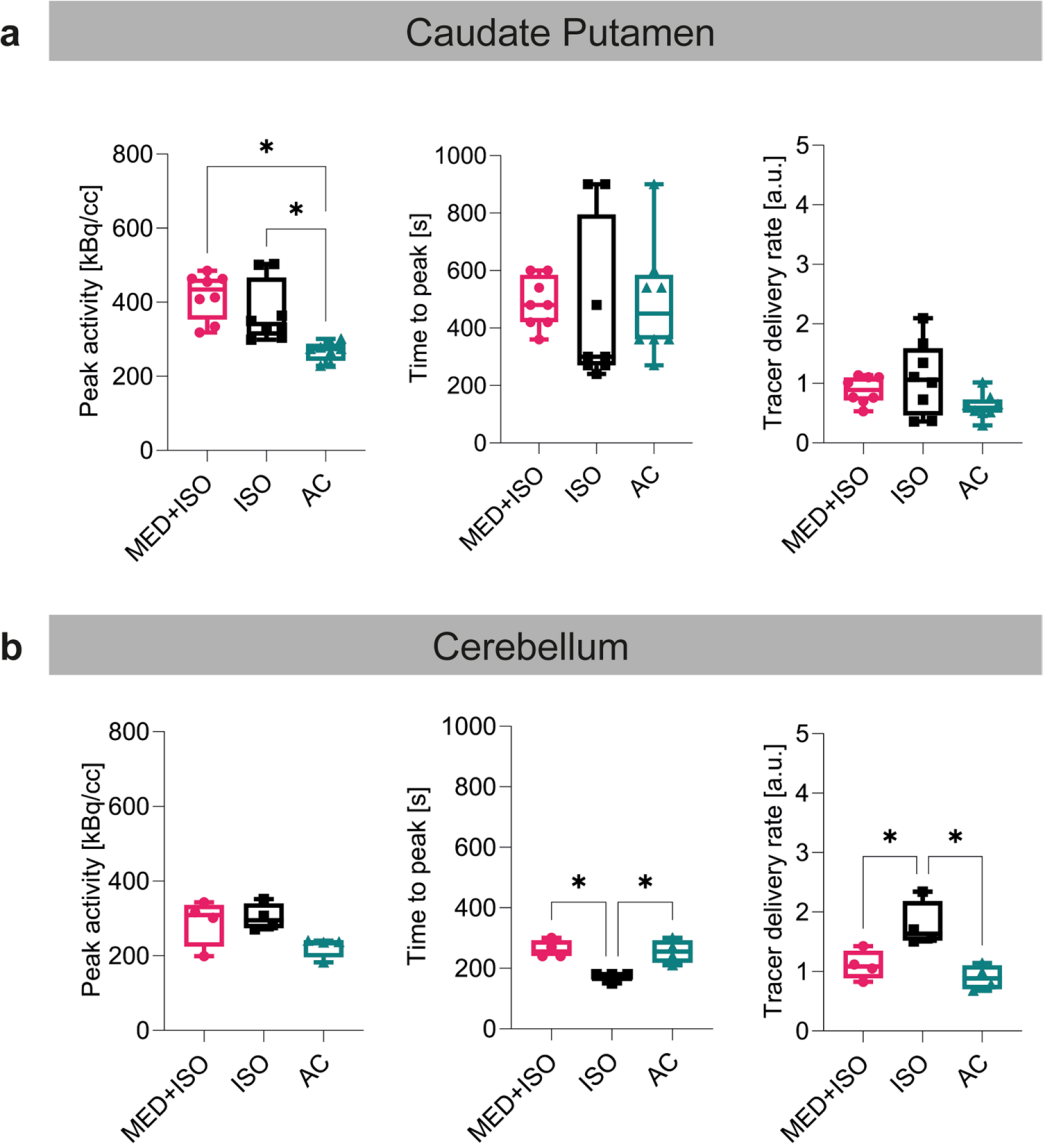
Effect of anesthesia with ventilation and pancuronium infusion on [^11^C]raclopride kinetics. **a** Peak activity, time to peak and tracer delivery rate of [^11^C]raclopride illustrated as box plots indicating the mean and standard deviation for the caudate putamen and (**b**) the cerebellum for group 3 (0.5% isoflurane and medetomidine), group 4 (1.3% isoflurane) and group 5 (α-chloralose). *p ≤ 0.05. Abbreviations: MED: medetomidine, ISO: 1.3% isoflurane, AC: α-chloralose

Peak [^11^C]raclopride activity, time to peak, and tracer delivery rate of [^11^C]raclopride for the CPu and the cerebellum under different anesthesia protocols with mechanical ventilation and pancuronium bromide infusion are illustrated in the Fig. 5 and Table S2. For more detailed results, refer to the supplementary material.

## Discussion

Neuroimaging has evolved significantly over the years, with methodologies like PET and fMRI becoming increasingly integrated. Such advancements have brought about novel challenges and the need to optimize experimental protocols. A critical consideration in PET imaging studies is the timing of BP_ND_ (binding potential non displaceable) calculations relative to the equilibrium state of tracer activity in target and reference regions. Calculating BP_ND_ before equilibrium is achieved can introduce bias, particularly if the reference region stabilizes earlier than the target region, leading to potential underestimation of the BP_ND_. In our study, the time-activity curves (Figs. 2 and 4) demonstrate that ligand concentrations in both the striatal and cerebellar regions continue to change within the 60-min acquisition window, with SUVR curves exhibiting a continuous rise. While the MED-V-P and 1.3% ISO+V+P groups may approach equilibrium after approximately 50 min, the lack of a fully stable equilibrium state within this timeframe highlights the limitation of this protocol. Extending PET scan durations to 90 min or longer would likely provide more reliable BP_ND_ estimates by ensuring that both target and reference regions reach a true equilibrium state. Future studies should consider this adjustment to minimize potential bias in BP_ND_ calculations. Using bolus plus constant infusion PET tracer protocols, it is possible to examine stimulus-related brain activation during one PET measurement [27, 28]. This tracer administration protocol enables a prolonged presence of the tracer in plasma and tissue, thereby facilitating the application of pharmacological and optogenetic stimuli in PET or PET/fMRI studies [5, 10]. However, the application of this protocol in [^11^C]raclopride PET imaging studies requires a fast equilibrium state between the target and reference region to enable a baseline and stimulation measurement during a 90-min PET acquisition. The presented study comprehensively examines the intricate relationships between mechanical ventilation and anesthesia protocols and their subsequent effects on [^11^C]raclopride PET kinetics in rat brains. Central to our findings is the profound effect of mechanical ventilation on [^11^C]raclopride tracer kinetics. While mechanical ventilation is frequently used in anesthetized fMRI experiments to mitigate motion artifacts [29] and regulate pCO_2_ levels to prevent hypercapnia [30, 31], its implications for PET imaging remains underexplored. Our study demonstrates that when using [^11^C]raclopride PET imaging under mechanical ventilation, the equilibrium state is achieved much later than under spontaneous breathing conditions, making stimulation experiments unfeasible. This delay likely stems from a decrease in CBF attributed to lower pCO_2_ levels [18]. This hypothesis is supported by a study from Sander and colleagues who examined CBF alterations in PET studies with [^11^C]raclopride and [^18^F]fallypride using simultaneous PET/MRI in baboons [32]. Their findings highlighted a notable increase in CBF following hypercapnic challenges with 7% CO_2_, which did, however, not influence the tracer binding potential. The lack of arterial blood gas analysis in our study, especially missing pCO_2_ values during measurements, does limit the depth of our physiological interpretation. Nonetheless, the use of different anesthetics in our study did not counteract the observed effect of mechanical ventilation on the tracer kinetics in our investigation. Intubation in small animals, such as rodents, is challenging due to their narrow airways, small size and fragile tissues, which complicate the visualization and access of the trachea and larynx [18]. Successful intubation requires a thorough understanding of species-specific anatomy as well as precise technique and equipment to reduce trauma and ensure proper tube placement [33]. The use of small-diameter endotracheal tubes increases the risk of blockages from mucous plugs and tube kinking, particularly during prolonged imaging sessions. To mitigate these risks, it is crucial to perform regular suctioning, select the appropriate tube size and type, and continuously monitor throughout the procedure.

Different anesthetics can significantly influence CBF, cerebral metabolism, and receptor availability which are crucial for PET imaging. Isoflurane is a potent vasodilator and can lead to increased CBF, which may affect tracer delivery and distribution [30]. This alteration in blood flow could impact the quantification of receptor binding and overall tracer kinetics. Additionally, the depressive effect of isoflurane on cerebral oxygen metabolism and its effect on the dopaminergic system may influence the dynamics of receptor availability [34]. Other studies have linked sevoflurane concentration with altered time to peak radioactivity in [^11^C]raclopride PET imaging in non-human primates [35]. In another study by Hassoun et al., a significant increase in BP and CBF was observed under halothane anesthesia in the striatum of awake cats, with changes in CBF playing a key role in these effects. These findings align with our observations, where anesthesia protocols significantly impacted [^11^C]raclopride tracer kinetics, likely due to alterations in CBF [36]. Furthermore, fMRI investigations have suggested that specific dosages of isoflurane may obscure naturally established connections in the rat brain [19]. Medetomidine, in contrast, generally causes less pronounced vasodilation and maintains a more stable CBF compared to isoflurane. It also induces a moderate reduction in cerebral metabolism, which may be advantageous for studies requiring steady-state condition over longer periods. However, its effect on the release and turnover of dopamine could potentially influence D2 receptor availability and should be considered when interpreting PET data [37]. In our investigation focusing on tracer kinetics we applied a combination of a low dose isoflurane with medetomidine. While the molecular mechanisms underlying the combined effect of these two medications remain a subject of study, their efficacy in small animal fMRI studies has been well established [6, 22, 38]. When combined with isoflurane, medetomidine can partially counterbalance the vasodilation caused by isoflurane [22]. Additionally, the supplemental low dose of isoflurane can abolish epileptic activities from dexmedetomidine infusion and maintain robust neuronal and hemodynamic responses for up to three hours [39]. The quick recovery from isoflurane and reversibility of medetomidine by atipamezole render this combination particularly beneficial for longitudinal imaging studies. However, our data show that [^11^C]raclopride kinetics are reduced compared to isoflurane alone, shown in the increased time to reach pseudo-equilibrium. Virtanen further reported the inhibitory effects of medetomidine on the release and turnover of neurotransmitters, such as noradrenaline, dopamine, and serotonin, which has to be kept in mind when studying neurotransmitter release [37]. AC, frequently employed in functional neuroimaging, operates mainly by augmenting GABA-A receptor activity, reducing overall brain activity [30, 40]. While its potentially toxic effects limit its long-term use, AC offers a robust functional-metabolic connection [41, 42]. It facilitates robust fMRI BOLD responses, improving sensitivity to subtle stimuli like whisker stimulation [43]. Its maintenance of near-normal physiological parameters may lead to more reliable receptor availability measurements. In our [^11^C]raclopride PET imaging study, AC showed an overall decreased delivery of the tracer to the brain and slower washout kinetics.

## Conclusions

In conclusion, our study shows that mechanical ventilation primarily affects [^11^C]raclopride kinetics and leads to a substantial shift of the tracer equilibrium state, making it unfeasible for bolus plus constant infusion studies requiring early tracer equilibrium states between a target and reference region for early interventions. Furthermore, it should be noted that ventilating and immobilizing small animals is an intrusive procedure and may not be suitable for longitudinal experiments. Moreover, intubating animals presents considerable technical challenges, and complications like hemorrhage or blockage of the tracheal tube can arise during the measurement. Thus, the ease of imaging animals under spontaneous breathing renders it a viable option. For [^11^C]raclopride PET experiments, we recommend, based on our results, a combined anesthesia of 0.5% isoflurane and medetomidine (absolute bolus 0.04 mg and 0.15 mg/kg/h infusion) without mechanical ventilation if experiments are performed under resting state conditions. If stimulations of pharmacological challenges are applied during the measurement, mechanical ventilation will reduce motion artifacts, and thus, a low (1.3%) isoflurane anesthesia may present an acceptable compromise. Therefore, in simultaneous PET/fMRI experiments in small animals, the influence on the values of both modalities must be considered when selecting an appropriate ventilation and anesthesia protocol and should be carefully examined beforehand.

## Supplementary Information

Below is the link to the electronic supplementary material.

Supplementary file1 (DOCX 387 KB)

## Data Availability

The data will be made available upon request.
