# Supplementary material for: Impact of Mechanical Ventilation and Anesthesia on PET Tracer Kinetics for Combined PET/fMRI Studies in Rats
Source: Mol Imaging Biol. 2025 May 14;27(3):313–23. doi: 10.1007/s11307-025-02006-3 (PMC12162802; doi:10.1007/s11307-025-02006-3)
Supplement: Supplementary file 1 — Supplementary file1 (DOCX 387 KB) [file 11307_2025_2006_MOESM1_ESM.docx]

**Electronic Supplementary Material**

**Impact of Mechanical Ventilation and Anesthesia on**

**PET Tracer** **Kinetics for** **Combined PET/fMRI Studies in Rats**

**Journal: Molecular Imaging and Biology**

Yan Ma^1,2,3*^, Laura Kuebler^1,4,5*^, Sabrina Haas^1^, Andreas Maurer^1,4^, Kristina Herfert^1#^

^*^ contributed equally

^#^corresponding author

1. Werner Siemens Imaging Center, Department of Preclinical Imaging and Radiopharmacy, University Tuebingen, Tuebingen, Germany
2. Max Planck Institute for Biological Cybernetics, Tuebingen, Germany
3. Graduate Training Centre of Neuroscience, University Tuebingen, Tuebingen, Germany
4. Cluster of Excellence iFIT (EXC 2180) "Image Guided and Functionally Instructed Tumor Therapies", University of Tuebingen, Tuebingen, Germany
5. German Cancer Consortium (DKTK), DKFZ, Core Center Heidelberg, Heidelberg, Germany

**Corresponding author:**

Prof. Dr. Kristina Herfert

Werner Siemens Imaging Center, Department of Preclinical Imaging and Radiopharmacy,

University of Tuebingen, Germany

Röntgenweg 13

Tübingen, Germany

Phone: +49 7071 29 87680

Fax: +49 7071 29 4451

E-Mail: kristina.herfert@med.uni-tuebingen.de

# Material and methods

**Radiotracer Synthesis**

The synthesis of [^11^C]raclopride was carried out based on the methodology outlined by Langer *et al.* [1] but utilized the refined high-performance liquid chromatography (HPLC) conditions proposed by van Laeken *et al.* [2]. After dilution with water, the separated fraction was concentrated on a conditioned Strata-X cartridge (Phenomenex, Aschaffenburg, Germany), eluted with 0.5 ml ethanol and diluted with 5 ml phosphate-buffered saline.

**Statistical Analysis**

The statistical analysis was conducted to assess the effects of respiratory conditions and anesthesia protocols on peak tracer activity, time to peak, and tracer delivery rate in [11C]raclopride PET imaging. Data that followed a normal distribution were analyzed using an unpaired t-test and ordinary one-way ANOVA, preceded by an F-test to determine variance equality. To account for multiple comparisons, a false discovery rate (FDR) correction was applied using the Two-Stage Linear Step-Up Procedure of Benjamini, Krieger, and Yekutieli (BKY) for post-hoc comparisons. A significance level of p ≤ 0.05 was used for all tests, with statistical computations performed using GraphPad Prism v9.1.0 (221).

# Results

**Effect of respiratory conditions on [^11^C]raclopride kinetics**

Differences in peak activity values were revealed in both the CPu and cerebellum across the three respiration conditions (CPu: p = 0.0178; Cerebellum: p = 0.0121). Post-hoc analysis showed that the MED-V-P condition had higher activity values compared to both MED+V-P (CPu: p = 0.0095; Cerebellum: p = 0.0065) and MED+V+P (CPu: p = 0.0189; Cerebellum: p = 0.0118). Differences in time-to-peak values were also observed in the CPu and cerebellum (CPu: p = 0.0043; Cerebellum: p = 0.0001), with MED-V-P values higher than those of MED+V-P (CPu: p = 0.0184; Cerebellum: p = 0.0001) and MED+V+P (CPu: p = 0.0014; Cerebellum: p < 0.0001). Differences in tracer delivery rates were noted in both the CPu and cerebellum (CPu: p = 0.0009; Cerebellum: p = 0.0001). Post-hoc tests indicated that MED-V-P showed higher delivery rates compared to MED+V-P (CPu: p = 0.0017; Cerebellum: p = 0.0001) and MED+V+P (CPu: p = 0.0005; Cerebellum: p = 0.0001).

**Effect of different anesthetics on [^11^C]raclopride kinetics**

Differences in peak activity values within the CPu was observed across the three anesthesia protocols (p = 0.0003). Post-hoc analysis revealed that the AC condition exhibited lower activity values compared to both the MED+ISO (p < 0.0001) and ISO (p = 0.0028) conditions. In contrast, no differences in peak activity values were found in the cerebellum among the anesthesia protocols (p = 0.0721). Additionally, there were no differences in time-to-peak values (p = 0.9381) or tracer delivery rates (p = 0.0778) in the CPu across protocols. However, in the cerebellum, differences were detected for both time-to-peak values (p = 0.0031) and tracer delivery rates (p = 0.0050). Post-hoc analysis indicated that the ISO protocol resulted in a shorter time-to-peak than both the MED+ISO (p = 0.0018) and AC (p = 0.0031) conditions and a faster tracer delivery rate compared to both MED+ISO (p = 0.0095) and AC (p = 0.0020).

1. Langer, O., et al., *Precursor synthesis and radiolabelling of the dopamine D-2 receptor ligand [C-11]raclopride from [C-11]methyl triflate.* Journal of Labelled Compounds & Radiopharmaceuticals, 1999. **42**(12): p. 1183-1193.

2. Van Laeken, N., et al., *Improved HPLC purification strategy for [C-11]raclopride and [C-11]DASB leading to high radiochemical yields and more practical high quality radiopharmaceutical formulations.* Applied Radiation and Isotopes, 2013. **78**: p. 62-67.

**Supplementary Tables**

**Table S1: Peak [^11^C]raclopride activity, time to peak, and tracer delivery rate of [^11^C]raclopride for the CPu and the cerebellum under different respiratory conditions.**


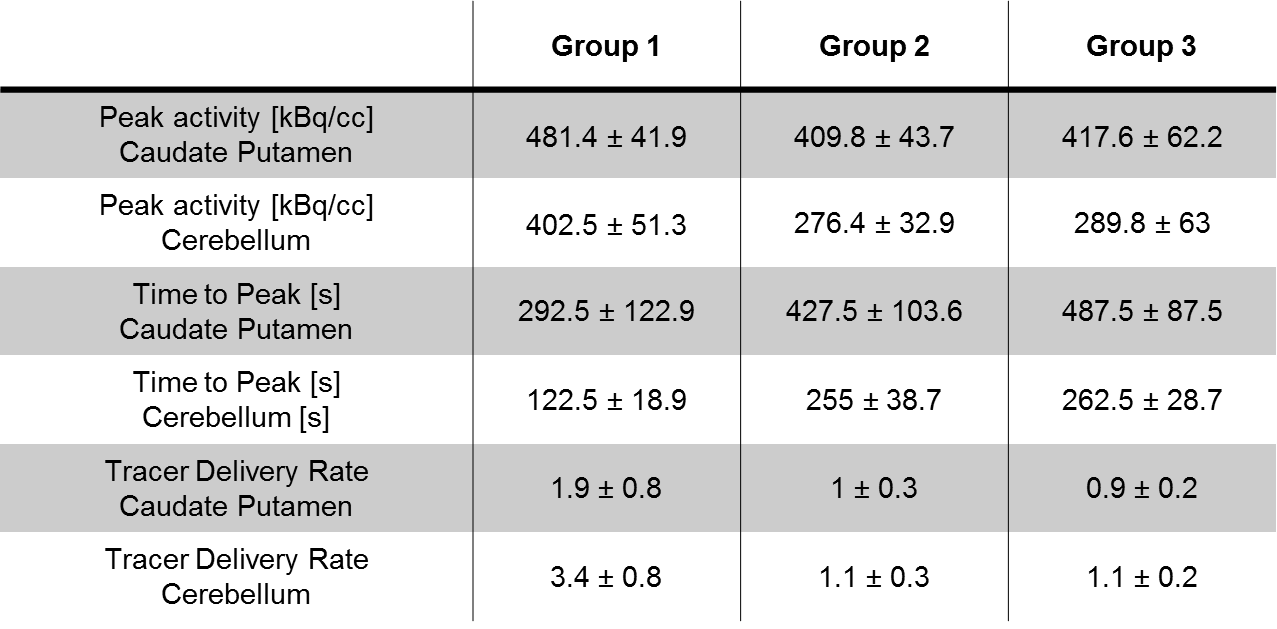


**Table S2: Peak [^11^C]raclopride activity, time to peak, and tracer delivery rate of [^11^C]raclopride for the CPu and the cerebellum under different anesthesia protocols**


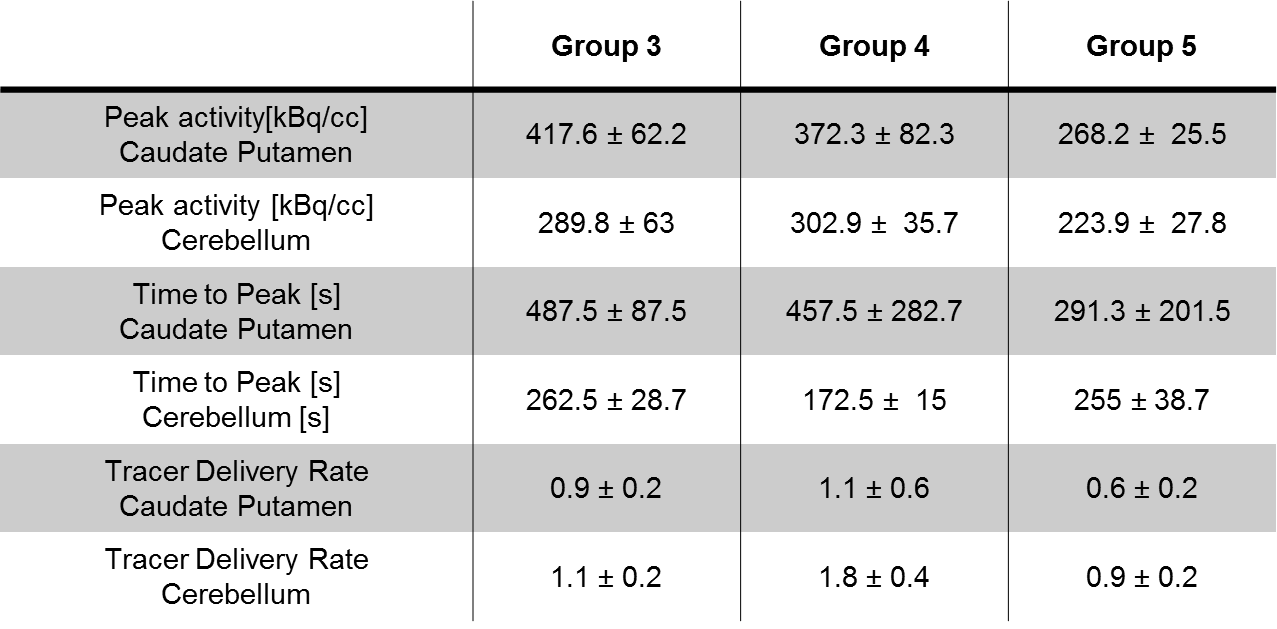


**Supplementary Figure**

**
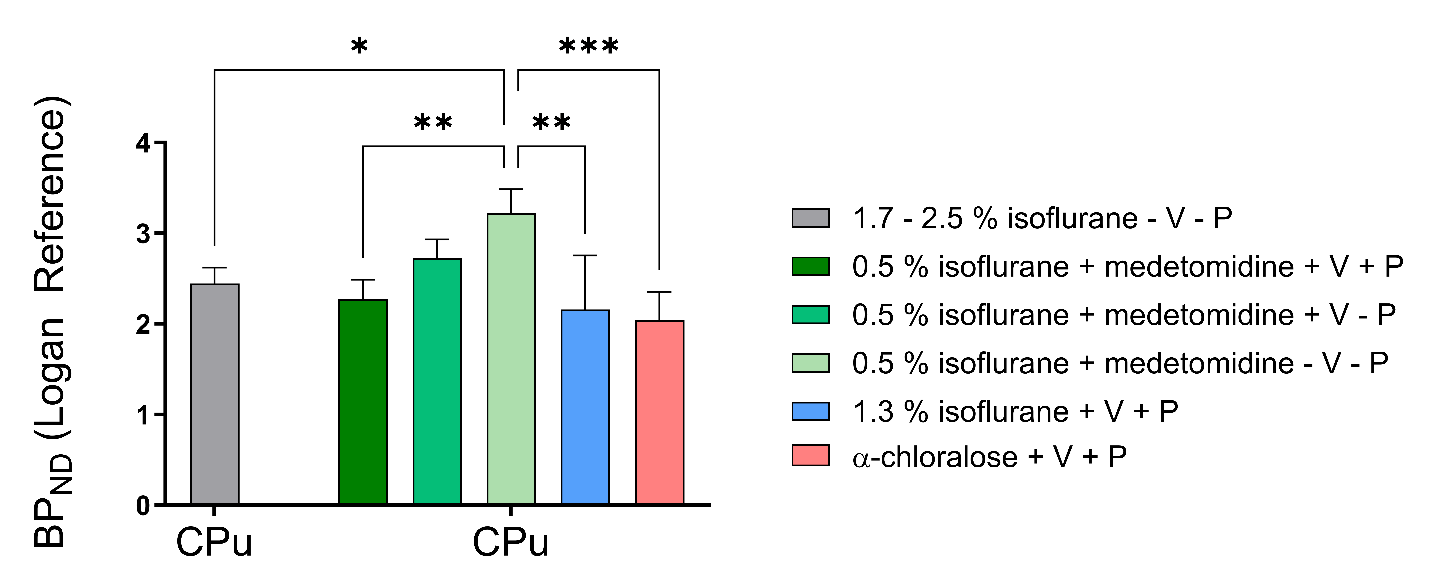
**

**Figure S1:** BP_ND_ of [^11^C]raclopride for the caudate putamen (CPu) calculated with the Logan Reference Model using the cerebellum as reference region compared to the rats under 1.7-2.5% isoflurane (grey bar) using a t* of 30 minutes.
